# Effectiveness of Folic Acid Fortified Flour for Prevention of Neural Tube Defects in a High Risk Region

**DOI:** 10.3390/nu8030152

**Published:** 2016-03-09

**Authors:** Haochen Wang, Hans De Steur, Gong Chen, Xiaotian Zhang, Lijun Pei, Xavier Gellynck, Xiaoying Zheng

**Affiliations:** 1 Institute of Population Research/WHO Collaborating Center on Reproductive Health and Population Science, Peking University, Beijing 100871, China; woody_37@126.com (H.W.); chengong@pku.edu.cn (G.C.); windson_zxt@126.com (X.Z.); peilj@pku.edu.cn (L.P.); 2 Department of Agricultural Economics, Faculty of Bio-Science engineering, Ghent University. Ghent 9000, Belgium; Hans.DeSteur@UGent.be (H.D.S.); Xavier.Gellynck@UGent.be (X.G.)

**Keywords:** community intervention, folic acid fortified flour, neural tube defects, DALY, China

## Abstract

Despite efforts to tackle folate deficiency and Neural Tube Defects (NTDs) through folic acid fortification, its implementation is still lacking where it is needed most, highlighting the need for studies that evaluate the effectiveness of folic acid fortified wheat flour in a poor, rural, high-risk, NTD region of China. One of the most affected regions, Shanxi Province, was selected as a case study. A community intervention was carried out in which 16,648 women of child-bearing age received fortified flour (eight villages) and a control group received ordinary flour (three villages). NTD birth prevalence and biological indicators were measured two years after program initiation at endline only. The effect on the NTD burden was calculated using the disability-adjusted life years (DALYs) method. In the intervention group, serum folate level was higher than in the control group. NTDs in the intervention group were 68.2% lower than in the control group (OR = 0.313, 95% CI = 0.207–0473, *p* < 0.001). In terms of DALYs, burden in intervention group was approximately 58.5% lower than in the control group. Flour fortification was associated with lower birth prevalence and burden of NTDs in economically developing regions with a high risk of NTDs. The positive findings confirm the potential of fortification when selecting an appropriate food vehicle and target region. As such, this study provides support for decision makers aiming for the implementation of (mandatory) folic acid fortification in China.

## 1. Introduction

Fifty years after the first indications of the potential role of folic acid in pregnancy [1] and nearly 40 years after groundbreaking work on folic acid as a means of preventing neural tube defects (NTDs) [2], the scientific evidence underpinning the effectiveness of this vitamin [3,4] is considered among the strongest in the field of congenital anomalies [5]. Unfortunately, the call for folic acid intervention programs, either by promoting folic acid supplements or by introducing a fortification policy, has been only partially addressed. Folate deficiency and, thus, NTDs (e.g., spina bifida, anencephaly and encephalocele) are still widely prevalent in various parts of the world [6], highlighting the need for implementing improved, tailored and effective prevention programs [7]. Although folic acid supplementation was demonstrated as effective in the RCTs (Randomized Controlled Trial) that established the preventive role of folic acid in NTDs, due to its undoubted effect on first occurrence [8] as well as recurrence [9], fortification of foods with folic acid has become more important. Despite its more recent introduction—the first national scale fortification of flour with folic acid in Oman [10] and the first mandatory wheat fortification programs were established in 1998 in the United States and Canada—various before-and-after studies have demonstrated a declining trend in NTD prevalence in the post-fortification period [11], resulting in similar risk reductions to those found with folic acid supplementation [12]. Moreover, case studies in Chile [13], South Africa [14], the USA [15], Australia and New Zealand [16] proved that adding folic acid to flour is also a cost-effective means to alleviate the burden of NTDs.

According to Bell *et al.* [17], approximately 1.75 billion people in the world have consumed folic acid fortified wheat flour, resulting in a market share of 29.3% of the worldwide flour consumption in 2008. Following the successful and effective implementation of folic acid fortified grains, many researchers indicated the importance of and need for similar mandatory or voluntary fortification programs in other developed regions such as Oceania [18] and Europe [19,20], and they especially emphasized the greater effectiveness of mandatory policy compared with voluntary policy [21] However, from a health perspective, poor developing regions need to become target areas of fortification because their populations depend on only one or a few staple crops and, as a consequence, they consume a lower level of daily folate than the level needed to prevent NTDs in pregnancy (below 400 μg per day) [22,23]. Moreover, as demonstrated for Canada, folic acid fortification’s impact on folate intake levels and NTD prevalence is larger in regions where the NTD prevalence in the pre-fortification period was relatively high [11,14].

The objective of this paper is to describe the study. The objective of the study is to evaluate the effectiveness of fortified floor in a poor, rural, high-risk NTDs region of China, namely Shanxi Province. Situated in northern China, this wheat-consuming region has one of the highest NTD birth prevalences in the world, as 138.70 per 10,000 pregnancies [24], 149.00 per 10,000 pregnancies [25] or even 199.38 per 10,000 pregnancies [26]. Not surprisingly, this region is also characterized by suboptimal folate intake levels. Nearly 44% of all pregnant women in Shanxi Province do not reach the recommended nutrient intake level [27] because of extremely low compliance with folic acid supplements (10%) [28] and low folate content in their current (non-fortified) diet [29]. Although community intervention studies in Shanxi [30] and other Chinese provinces [31] lend support to folic acid supplementation, the effectiveness of the free distribution of folic acid supplements to reduce the prevalence of NTDs disappeared as soon as the program ceased [32].

Although Shanxi is by far the most problematic province of China [33,34], folate deficiency and NTDs are a major public health problem in the whole country and particularly in its northern provinces. If the threshold of folate deficiency is defined by a plasma folate concentration below 10 Nmol/L, China has a folate deficiency prevalence of approximately 9.3% among women of childbearing age and 19.6% among the general population with an annual number of 12,000 folate-related NTDs, so China’s burden of folate deficiency is among the world’s largest, both in relative and absolute terms [35].

To measure the effectiveness of folic acid fortified wheat flour, this paper used data based on a community intervention study. The focus is on the birth prevalence of NTDs as the main adverse health outcome of folate deficiency. This allows the application of the disability-adjusted life years (DALYs) framework to quantify the benefits, in line with previous health effect assessments of folate interventions [36].

## 2. Experimental Section

### 2.1. Study Design

The community intervention study was carried out in three counties in Shanxi Province, authorized by the National Population and Family Planning Commission. Eleven villages were random selected: eight villages from Zhongyang and Jiaokou Counties, representing the intervention group, and three villages from Liulin County, representing the control group. These three counties are located in the same region, and the villages were selected randomly using cluster sampling. To avoid biases due to regional dietary patterns, a baseline dietary survey was carried out from 2002 to 2004 in the three selected counties. It indicated that the control and intervention group had similar dietary patterns, with the same staple food, wheat flour [37]. The daily intakes of dietary folate in Zhongyang, Jiaokou and Liulin were only 53.6%, 66.5%, and 42.1% of RNI (Recommended Nutritional Intake) [37], which is 400 μg/day [38], respectively. As expected, the dietary survey confirmed that the intake of various nutrients is still insufficient, especially that of folate [29].

In the present study, all women who were preparing for marriage or planning a pregnancy were included in this project by a family planning network. In them, women who would marry from January 2006 to December 2007 and who were planning a pregnancy at some point between 1 January 2006 and 31 December 2007 were identified as eligible participants. Dietary data were collected as part of this study with a semi-quantitative food frequency questionnaire which had been validated [39]. This study obtained the ethical approval which was given by the medical ethics committee of Institutional Review Board of Peking University with the following reference number: IRB00001052-08083. Workers of Shanxi Population and the Family Planning Commission (PFPC) helped the participants to familiarize themselves with the project and its goals. All participants signed the informed consent forms. The women of child bearing age who consumed fortified flour in this study (intervention group) accounted for 33.4% of all women of child-bearing age in the counties where the fortified flour was distributed. At the beginning, 16,811 women were recruited as the intervention group and 7260 women were recruited as the control group. Finally, after the exclusion of participants who left the village, lost contact or stopped consuming fortified flour at the endline (163 women in intervention group and 58 women in control group), 16,648 women in the intervention group, of which 9132 and 7516 women were in Zhongyang County and Jiaokou County, respectively and 7037, women in the control group, were used for analyses of flour consumption, NTD birth prevalence and DALYs calculation. For women who dropped this program, their pregnant outcomes were lost.

Women in the intervention group were asked to keep their regular dietary habits but change ordinary flour to fortified flour. In all villages of the intervention group, fortified flour was distributed to families with women of childbearing age (18–35 years old) in groups. Workers of Shanxi Population and the Family Planning Commission (PFPC) were responsible for the distribution of flour tickets (0.4 kg/day per person). It is important to state that the selected villages were not neighboring and that the Population and Family Planning Commission (PFPC) staff closely followed up the families who consumed fortified flour. Although no fortified flour was sold in the control areas, we cannot be certain that there was no unauthorized distribution of fortified flour. Nevertheless, based on the aforementioned methodological choices, such exchanges should be negligible (if present) [40].

Women in the intervention group were asked to record their approximate daily consumption of flour using the same questionnaire used in the baseline dietary survey [37]. Every month, workers validated these records through face-to-face interviews with these mothers and then calculated the monthly flour consumption of the respondents. Regarding the pregnant outcomes, clinicians went to all villages of Zhongyang and Jiaokou to perform a physical examination of all infants (normal and abnormal) at a makeshift clinic for the diagnosis of NTDs and other diseases. The staff of the Population and Family Planning Commission (PFPC) recommended all women go to a hospital when giving birth. Very few babies were born at home and they were examined by doctors. Scholars in the research team followed up every month to record pregnant outcomes, including spontaneous abortion, induced abortion, fetal death, still birth, live birth and occurrence of birth defects. The time span of fortified wheat flour consumption amounted to 24 months (from 1 January 2006 to 31 December 2007).

The fortified flour, distributed in intervention villages, consisted of a mix of micronutrients; in addition to folic acid (2 mg/kg), it was fortified with Vitamin B1 (3.5 mg/kg), Vitamin B2 (3.5 mg/kg), iron (30 mg/kg) and zinc (25 mg/kg). This fortification was in line with WHO recommendations of folic acid fortification for flour [41]. Samples of fortified flour were tested to ensure they were fortified with folic acid adequately following the standard of GB/T (National Standard/Recommendation) 5009-2003 and recommended methods [42] by the County Grain Bureau.

This study was a cross-sectional survey completed two years after the fortification project was initiated. Furthermore, there were no baseline data on NTDs or biological indicators. In this study, we used descriptive statistics to illustrate the demographic characteristics, and used *t*-test and one-way ANOVA to compare differences between the intervention group and control group. The software for statistics was SPSS 19.0 (SPSS Inc., New York, NY, USA, 2000).

### 2.2. Analysis of Biological Indicators

By 31 December 2007, various biological indicators had been monitored in each of the selected counties: serum folate and homocysteine. Through multi-stage random sampling, subjects who had delivered a baby were randomly selected; in the intervention group, women who consumed fortified flour for less than 12 months were excluded (*x* = 46).

### 2.3. Health Effect Assessment

Data collection regarding women who gave birth or had newborn babies was carried out between August 2006 and November 2007 to ensure women in the intervention group were able to consume enough fortified flour. Depending on their assignment (intervention *versus* control group), nearly all women delivered in one of the three counties’ hospitals. NTD cases in this study referred to anencephaly (Q00.0), spina bifida (Q05.9) and encephalocele (Q01.9), based on the ICD-10 code [25]. Although mothers in the intervention and control groups did not go to the same hospitals, their delivery care was similar. The clinicians of the hospitals in both groups were trained with the same standards. They were specifically trained to identify and record cases of NTDs and to ensure that all cases were reported to the project team. The doctors implemented prenatal diagnosis for birth defects. Stillbirths were also specifically checked to avoid missing cases. When a pregnant mother went to a clinic for a prenatal examination and there was no ultrasound available, they were recommended to go to a township health center to take the ultrasound examination.

According to one previous study, the quality control assessment after the survey of NTDs in this region, which indicated that only 3% of births were not captured, the number of missing cases in our community intervention does not deviate far from this value [25].

In addition to the reduction of NTD birth prevalence as a straightforward indicator of the actual health effect of folic acid fortification, we also applied the DALYs framework to evaluate the health benefits in terms of the total number of DALYs that can be saved through the introduction of fortified wheat flour. Initially introduced as a measure of disease burden, this method combines mortality and morbidity related information in a single index. We follow the approach of De Steur *et al.* [35,36] in folate and other micronutrient interventions by collecting and inserting various parameters into the DALY formula:

(1) $${\mathrm{DALYs}}_{\mathrm{lost}}=\underset{j}{\sum }T_{j}M_{j}\left(\frac{1-e^{-{\text{rL}}_{j}}}{r}\right)+\underset{j}{\sum }T_{j}I_{\text{ij}}D_{\text{ij}}\left(\frac{1-e^{-{\text{rd}}_{\text{ij}}}}{r}\right)$$

where T_j_ is the total number of people in target group j; M_j_ is the mortality rate related to NTD type (anencephaly, spina bifida and encephalocele) in target group j; r is the discount rate; Lj is the average remaining life expectancy for target group j; I_ij_ is the birth prevalence of NTDs i in target group j; D_ij_ is the disability weight for NTD type (spina bifida; encephalocele) i in target group j; and d_ij_ is the duration of NTD type (spina bifida; encephalocele)

When considering NTD prevention measures, such as fortified wheat flour, the target group refers to the type of NTDs: anencephaly (fatal) or spina bifida and encephalocele (fatal or non-fatal) [43]. D_ij_, which denotes disability weight, is 0.593 and 0.520 for spina bifida and encephalocele, respectively [44]. Types of NTDs in this study were fatal, leading to stillbirth, therefore, M_j_ equals 1 and L_j_ equals dij in this study. L_j_ is the life expectancy in Shanxi. According to the fifth census in 2000, the average life expectancy for people in Lvliang region, Shanxi Province was 71.65 years [45]. The discount rate r is 0.3.

The health effects can then be calculated by comparing the relative number of DALYs lost (per 10,000 births) in the control and intervention group.

## 3. Results

### 3.1. Demographic Characteristics

The differences of age between the intervention and control group are not statistically significant, as shown in Table 1. For the educational level, women in these two groups were not significantly different. According to monthly calculations, approximately 78.9% of women in the intervention group consumed more than 200 g per day, whereas 38.1% of women consumed more than 300 g per day.

### 3.2. Consumption of Fortified Flour

Table 2 summarizes the folate intake levels and flour consumption patterns in the control and intervention groups. The table shows women in the intervention group had more folic acid from fortified flour intake.

### 3.3. Biological Indicators

According to the method of analyzing biological indicators, blood samples of 217 women were taken to determine levels of serum folate and homocysteine. Of this sample, 155 women were recruited in the intervention area (75 in Zhongyang and 80 in Jiaokou), compared to 62 women in the control group.

At the end of the study, women in the intervention group had substantially higher levels of serum folate than women in the control group (Table 3). In the case of fortified flour consumption, the mean serum folate level was 23.84–27.15 nmol/L. As expected, the homocysteine level was lower (11.31–14.25 μmol/L) in the intervention group compared with the control group. The differences between the two groups in our intervention study are significant (*p* < 0.001).

### 3.4. Differences in NTD Birth Prevalence

By comparing the occurrence of NTDs, fatal and non-fatal, in the control and intervention group, significant differences between them are shown (Table 4). Although the control group showed approximately 49 out of 2139 pregnancies to be affected by NTDs, the intervention group had a figure of 43 out of 5898 pregnancies. As such, fortified flour consumption lowered the birth prevalence of NTDs from 229.1 NTDs per 10,000 births (control group; reference) to 72.9 NTDs per 10,000 births (intervention groups; OR = 0.313, 95% CI: 0.207–0.473, *p* < 0.001). The birth prevalence in Zhongyang county, one of the counties of our intervention group, was 78.3 per 10,000 births (OR = 0.337, 95% CI: 0.209–0.544, *p* < 0.001). The birth prevalence in Jiaokou county, the other county of our intervention group, was 65.9 per 10,000 births (OR = 0.283, 95% CI: 0.163–0.493, *p* < 0.001).

### 3.5. Burden of NTDs and Health Effects (DALYs Saved)

Application of the DALY framework compares the burden of the different types of NTDs in both groups (Table 5). The total number of DALYs lost in the intervention group, *i.e.*, 1266 DALYs in two years, is relatively close to that in the control group (1107 DALYs in two years) except for the lack of non-fatal outcomes. Due to demographic group differences (e.g., birth rate, population size), the burden of NTDs is weighted according to the number of births. This relative burden of NTDs, 2146.98 DALYs, expressed in DALYs lost per 10,000 births in these two years, is substantially lower in the intervention group than in the control group. Comparison of both relative measures demonstrates that the introduction of folic acid flour is associated with an annual burden that is 58.52 % (1514.66 DALYs per 10,000 births per year) lower than in a controlled setting.

## 4. Discussion

This study reports the results of an intervention study on the effectiveness of fortified wheat flour as a potential health strategy to tackle the burden of folate deficiency in high-risk regions of NTDs. Building on a previous study, the Chinese Shanxi Province was taken as a relevant case. Often named one of the world’s most NTD-stricken regions, Shanxi is often targeted as a research location to introduce or examine the potential of different folate programs. This NTD-stricken status, as well as the low compliance and impacts of past folic acid supplementation programs, highlight the need for alternative efforts to improve folate intake levels, particularly among women of childbearing age.

By using a community intervention approach and selecting participants from regions, we could reduce the impact of other factors that often appear in before-after studies, as mentioned; for instance, validity of birth defect data from birth certificates or failure to record all NTD-affected spontaneous abortions, in the US study on the folic acid fortification policy [46]. A recall bias is also unlikely to occur because the consumption of fortified flour was recorded by health workers monthly.

The results lend support to folic acid fortification of flour as an effective means of increasing folate intake levels and drastically reducing the birth prevalence and burden of NTDs in a developing high-risk region, such as Shanxi Province. The analysis of biological indicators, such as serum folate and homocysteine, is a first but important indication of the potential of this folic acid fortification strategy, with similar results to another Chinese (case-control) study, in which the median of serum folate and homocysteine levels in women with healthy newborn babies was 26.29 nmol/L and 9.71 μmol/L, respectively [47]. However, the substantial discrepancy in NTD birth prevalence between the control and intervention groups (−68%) definitely shows that large health effects can be generated, which are in the magnitude of those found in analyses in various developed countries.

In the present study, the high prevention rate approximates the results of other folic acid supplementation programs in China. In studies of folic acid supplementation in Shanxi, China by Berry *et al.* [30], the reduction in NTD birth prevalence varied between 70% (low compliance) and 85% (high compliance). In other Chinese provinces, reduction rates of 80% were reported [31]. These figures are in line with those of studies in developed countries, where 73% of NTDs were prevented [48]. In studies on folic acid fortified food in other countries, the reduction of birth prevalence of NTDs includes 31% in the US [49], 40% in Chile [50], and 63% in rural areas of Costa Rica [51], which are substantially lower than results obtained through supplementation.

One should be careful when interpreting these results because even though women in the control group could not consume fortified flour, they may have been aware of the benefits of folic acid and, hence, paid attention to food or nutrition recommendations, which could have affected their serum folic acid levels and the birth prevalence of NTDs. Another often neglected factor is the compliance of consuming folic acid fortified foods. Although the scientific community has established a strong relationship between maternal, periconceptional folic acid supplementation and the reduction of NTDs, the success of such programs is often tempered by the lack of interest, knowledge and compliance of pregnant women to take a folic acid pill every day one month before and three months after conception. When looking at folate biofortification, in which the natural folate concentrations in staple foods are enhanced, and at folate biofortified rice in particular, the burden of NTDs could be reduced by 37.0% (low compliance) and 81.8% (high compliance) in the case of staple foods in northern China [35]. This shows the importance of compliance. The purpose of a fortification program is to remove the need for compliance by providing folic acid in the foods that the women already eat.

The acceptance of fortified flour, as well as the aforementioned effects of fortified flour consumption on key biological indicators and the burden of folate deficiency and NTDs, underline the need to select the most appropriate food vehicle for fortification when tackling a micronutrient deficiency.

Whereas our DALY figures correspond to those of studies using folate biofortified rice in Shanxi Province with low compliance [35], flour is the main staple crop in this region [37], which may explain the high compliance to fortified flour consumption.

There are some limitations in this study. One is the lack of baseline assessment of NTDs or biological indicators. This might weaken the effect of the results. However, four years before this study, there was a survey about NTDs in this region and these three counties had very high and similar birth prevalence of NTDs before intervention [25], which could be regarded as the base condition of NTDs in this region. These three counties also had similar dietary habits, nutrients intake and lower mean values of daily folate acid intake than the RNI [37]. The dietary habits were maintained until the end of this study. After intervention, the birth prevalence of NTDs in the intervention group decreased, which could prove the effectiveness of folic acid fortified flour. In the future, we should pay more attention to measuring more aspects of samples in the control group in order to make the two groups more similar. Another limitation is that the time we allocated to conducting the case-control study was a little short. Before the present study, the research team had conducted a long term study on the prevalence of NTDs. Then, to see whether provision of folic acid fortified flour has an effect on it, the research team carried out this study. Future research should be conducted over a longer period of time in order to obtain more information.

Nevertheless, future research is needed to determine whether this approach is also a cost-effective strategy to tackling folate deficiency in high risk regions of NTDs, such as Shanxi. Although previous studies in other regions confirm the potential cost-effectiveness of folic acid fortified foods [13,52,53], future research is needed to examine whether it is also the most suitable strategy, in terms of both acceptance and economic feasibility. Although China currently focuses its folic acid recommendations for women of childbearing age on supplementation [54], a mandatory fortified flour policy is still under discussion. Since the (small) market introduction of folic acid fortified flour in 2004, a national standard has been awaiting governmental authorization [55]. In this context, the findings of our intervention study shed light on the potential health effects that a positive decision on mandatory fortification in China could generate, at least in high risk regions.

## 5. Conclusions

The result of this effectiveness evaluation study of fortified flour indicates that the intake of fortified flour in a poor, rural, high-risk, NTD region can effectively improve the condition of NTDs in this region. The serum folate of child-bearing aged women in the intervention group, with the intake of fortified flour, was higher than the control group. The birth prevalence of NTDs and an annual relative burden of NTDs (DALYs per 10,000 birth per year) in the intervention group were substantially lower than the control group. Further, these finding can be regarded as an important evidence for policy makers from developing regions and countries that are bothered by a high birth prevalence of NTDs but limited input in rural areas, to consider the implementation of folic acid and multi-nutrients fortified flour.

## Figures and Tables

**Table 1:** Demographic characteristics of the intervention group and the control group.

	Control Group	Intervention Group			*p* Value (Control and Intervention)
Regions	Liulin	Zhongyang	Jiaokou	Total	
Number of childbearing women	7037	9132	7516	16648	
Number of births	2139	3318	2580	5898	
Age in year (Mean ± SD)	26.3 ± 4.6	26.8 ± 5.4	25.9 ± 5.6	26.4 ± 5.2	*p* = 0.1803
Education (Number and Percentage)					*p* = 0.1257
Primary school or below	1203 (17.1)	1102 (12.1)	929 (12.4)	2031 (12.2)	
Junior high school	4060 (57.7)	5622 (61.6)	4550 (60.5)	10172 (61.1)	
Senior high school	1147 (16.3)	1567 (17.2)	1330 (17.7)	2897 (17.4)	
College or above	626 (8.9)	841 (9.2)	707 (9.4)	1548 (9.3)	

**Table 2:** Mean daily dietary folate intake in the control and intervention group (before and after the fortification).

	Control Group	Intervention Group		
Regions	Liulin	Zhongyang	Jiaokou	Total
Number of childbearing women	7037	9132	7516	16,648
**Before Intervention**				
Mean consumption of wheat flour (g/day)	309.2 ^1^	356.5 ^1^	464.1 ^1^	405.1
Mean dietary folate intake (μg/day)	168.3 ± 72.1 ^1^	214.2 ± 65.6 ^1^	265.9 ± 89.7 ^1^	237.5 ± 76.4
**After Intervention**				
Mean flour consumption (g/day)	314.8 ± 46.2	362.7 ± 63.7	423.5 ± 57.4	390.1 ± 60.9
Mean folic acid intake from fortified flour (μg/day)	0.00	725.4 ^2^	847.0 ^2^	780.3

^1^ Based on [37]. ^2^ Based on the amount of folic acid in fortified flour (200 μg per 100 gram).

**Table 3:** Key bio-indicators in the control groups and intervention groups at endline.

	Control Group	Intervention Group			*p*-Value (Control and Intervention)
Regions	Liulin	Zhongyang	Jiaokou	Total	
*n*	62	75	80	155	
Serum folate (nmol/L) (Mean ± SD)	18.73 ± 5.47	27.15 ± 7.60	23.84 ± 7.83	25.44 ± 7.72	*p* < 0.001
Homocysteine (μmol/L) (Mean ± SD)	21.51 ± 11.90	14.25 ± 8.55	11.31 ± 6.03	12.73 ± 7.25	*p* < 0.001

**Table 4:** Effectiveness of the fortified flour intervention (24 months) at endline.

	Control Group	Intervention Group		
Regions	Liulin	Zhongyang	Jiaokou	Total
Births	2139	3318	2580	5898
NTDs	49	26	17	43
Fatal ^a^	41	26	17	43
Spina bifida	15	11	6	17
Encephalocele	16	6	7	13
Anencephaly	10	9	4	13
Non-fatal	8	0	0	0
Spina bifida	6	0	0	0
Encephalocele	2	0	0	0
Birth prevalence (NTDs per 10,000 births) ^b^	229.1	78.3	65.9	72.9
Odds Ratio	*Reference group*	0.337 (95% CI: 0.209–0.544)	0.283 (95% CI: 0.163–0.493)	0.313 (95% CI: 0.207–0.473)

^a^ Three main types of NTDs (spina bifida, anencephaly and encephalocele); ^b^ CI = Confidence Interval.

**Table 5:** Effects of fortified flour on the burden of NTDs.

	Control Group	Intervention Group		
Regions	Liulin	Zhongyang	Jiaokou	Total
Number of births	2139	3318	2580	5898
Burden of NTDs (DALYs lost)				
Total	1107.21	765.66	500.63	1266.29
Fatal outcomes	971.80	765.66	500.63	1266.29
Spina bifida	441.73	323.93	176.69	500.63
Encephalocele	471.18	176.69	206.14	382.83
Anencephaly	294.49	265.04	117.79	382.83
Non-fatal outcomes	134.48	0	0	0
Spina Bifida	97.29	0	0	0
Encephalocele	37.19	0	0	0
Relative burden of NTDs (Total DALYs lost per 10,000 births)	5176.29	2307.60	1940.41	2146.98
Difference between control group and intervention group (Total DALYs lost per 10,000 births)				−3029.31
Difference between control group and intervention group (DALYs lost per 10,000 births per year)				−1514.66

## Supplementary Materials

The following are available online at http://www.mdpi.com/2072-6643/8/3/152/s1, Figure S1. The flow chart of the study design.
