# Supplementary material for: Effectiveness of Folic Acid Fortified Flour for Prevention of Neural Tube Defects in a High Risk Region
Source: Nutrients. 2016 Mar 9;8(3):152. doi: 10.3390/nu8030152 (PMC4808880; doi:10.3390/nu8030152)
Supplement: Supplementary file 1 [file nutrients-08-00152-s001.docx]

Effectiveness of Folic Acid Fortified Flour for Prevention of Neural Tube Defects in a High
Risk Region

Haochen Wang, Hans De Steur, Gong Chen, Xiaotian Zhang, Lijun Pei, Xavier Gellynck and Xiaoying Zheng

**Figure S1.** The flow chart of the study design.

Purpose: To clarify for the reader the sample size used for NTDs, flour consumption and DALYs and those used for biological indicators. This flow chart is added as a figure to be posted as an
on-line supplementary material.
